# Differences in Cortical Sources of the Event-Related P3 Potential Between Young and Old Participants Indicate Frontal Compensation

**DOI:** 10.1007/s10548-016-0542-y

**Published:** 2017-01-18

**Authors:** R. van Dinteren, R. J. Huster, M. L. A. Jongsma, R. P. C. Kessels, M. Arns

**Affiliations:** 1 Research Institute Brainclinics, Nijmegen, The Netherlands; 2 Donders Institute for Brain, Cognition and Behavior, Radboud University Nijmegen, Nijmegen, The Netherlands; 3 Department of Psychology, University of Oslo, Oslo, Norway; 4 Psychology Clinical Neurosciences Center, University of New Mexico, Albuquerque, NM USA; 5 Behavioural Science Institute, Radboud University Nijmegen, Nijmegen, The Netherlands; 6 Department of Experimental Psychology, Utrecht University, Utrecht, The Netherlands; 7 Department of Medical Psychology, Radboud University Medical Center, Nijmegen, The Netherlands

**Keywords:** EEG, ERP, Aging, P3, P300

## Abstract

The event-related P3 potential, as elicited in auditory signal detection tasks, originates from neural activity of multiple cortical structures and presumably reflects an overlap of several cognitive processes. The fact that the P3 is affected by aging makes it a potential metric for age-related cognitive change. The P3 in older participants is thought to encompass frontal compensatory activity in addition to task-related processes. The current study investigates this by decomposing the P3 using group independent component analysis (ICA). Independent components (IC) of young and old participants were compared in order to investigate the effects of aging. Exact low-resolution tomography analysis (eLORETA) was used to compare current source densities between young and old participants for the P3-ICs to localize differences in cortical source activity for every IC. One of the P3-related ICs reflected a different constellation of cortical generators in older participants compared to younger participants, suggesting that this P3-IC reflects shifts in neural activations and compensatory processes with aging. This P3-IC was localized to the orbitofrontal/temporal, and the medio-parietal regions. For this IC, older participants showed more frontal activation and less parietal activation as measured on the scalp. The differences in cortical sources were localized in the precentral gyrus and the parahippocampal gyrus. This finding might reflect compensatory activity recruited from these cortical sources during a signal detection task.

## Introduction

The P3 (or P300) is a positive-going event-related potential recorded in the electroencephalogram that peaks at approximately 300 milliseconds after stimulus presentation (Sutton et al. 1965). The P3 that is elicited in an auditory signal detection task, i.e. the oddball paradigm (Ritter and Vaughan 1969), presumably reflects overlapping activity of several cognitive processes. It is generated by neural activity of multiple cortical structures (Friedman 2003).

### The Aging P3

The multifariousness of the P3 and the fact that it is affected by age-related changes in cognitive capacities, suggests it as a sensitive metric for cognitive performance (Van Dinteren et al. 2014b; Polich 1996). In an earlier study the developmental trajectories of the P3 amplitude across the lifespan were compared for frontal and parietal electrode sites. It was demonstrated that age-related changes were remarkably different between these locations. The parietal P3 amplitude increased in childhood to reach its peak in adolescence, then declined for the rest of the lifespan. In contrast, the frontal P3 amplitude reached its peak at a much older age, 46 years, after which it remained constant for the rest of the lifespan. It was concluded that the P3 wave reflects different mixes of cortical activation depending on the location of measurement and the age of the participant, among other probable factors. The frontal P3 amplitude might reflect compensatory activity from frontal regions that becomes more prominent as people age (Van Dinteren et al. 2014a).

This compensatory activity is possibly originating from the prefrontal cortex (PFC) that is related to higher order cognitive processes involved in regulating attention, working memory, and problem solving. These processes can be lateralized and it has been demonstrated that in older participants this lateralization reduces (this model is referred to as HAROLD, Hemispheric Asymmetry Reduction in Old Adults (Cabeza 2002)). This phenomenon might reflect changes in cognitive strategies used by older adults and/or neuroplastic reorganization in the aging brain (Cabeza et al. 2002). Similarly, the Compensation-related Utilization of Neural Circuits Hypothesis (CRUNCH) by Reuter-Lorenz and Cappell (2008) posits that the aging brain, facing a decline in cognitive performance, can compensate to some degree by increasing activity in alternative connected neural networks (Daffner et al. 2011; Cappell et al. 2010). The P3 potential has a relatively long duration and consists of multiple overlapping slow potentials that reflect the several cognitive processes that are devoted to the task at hand. In line with this thought, there are many neural generators identified for the P3, e.g. the PFC, the temporo-parietal junction, primary auditory cortex, and other sources (Friedman 2003). The P3 might be exceptionally sensitive to pick up subtle changes in the relative contribution of the different cognitive processes that give rise to it. Therefore, it is a likely candidate for capturing compensatory activity that is increasingly allocated as people age. A simple comparison of P3 potentials between older and younger adults would be a first step. However, one has to extract and separate the various underlying sources of information that altogether give rise to the P3 potential in order to compare both age groups thoroughly.

Besides the often reported anterior shift in topography of the P3 in aging (Li et al. 2013; West et al. 2010; Friedman et al. 1997; O’Connell et al. 2012), Frodl et al. (2000) observed two subcomponents of the P3 by dipole source analysis that were differently affected by aging. The temporo-parietal P3 subcomponent was smaller in older adults whereas the frontal P3 subcomponent remained unchanged. These findings seem to explain the noted topographical change of the P3 with aging. Furthermore, there are reports of differences between younger and older adults in activation of the cortical regions that contribute to the P3 wave. For instance, it has been reported that P3s of older adults consist of more activation in prefrontal (O’Connell et al. 2012) and temporal regions compared to younger adults (Tsolaki et al. 2015; O’Connell et al. 2012).

### The Decomposed P3

Because of the spatio-temporal overlap of activity from different neural sources inherent to multichannel scalp EEG recordings, methods for data decomposition have been brought forward to recover and separate the underlying source activity patterns. Independent component analysis (ICA) or principal component analysis (PCA), as applied to data of single as well as multiple subjects, have become prominent tools to recover neural source patterns from various measurement modalities (e.g., Eichele et al. 2011; Huster et al. 2015). ICA achieves this by decomposing the original recordings such that the resulting sources (usually referred to as components) exhibit maximal statistical independence; PCA, on the other hand, results in merely uncorrelated component activity patterns. PCA for EEG has a somewhat longer tradition, whereas studies applying ICA for the dissociation of P3 subcomponents are still somewhat scarce. Nonetheless, multiple studies have found independent components of this ERP using PCA and ICA that are physiologically plausible. Debener et al. (2005) decomposed concatenated single-trial ERP data from a novelty oddball paradigm and found two independent component clusters that respectively accounted for the novelty P3 and P3b responses. Brown et al. (2015) found a similar dissociation using PCA, and further reported a differential susceptibility of the two P3 subcomponents to drug exposure. Others also reported a dissociation of the frontal and parietal P3s from the succeeding slow wave activity that often appears as prolonged activity associated with the P3 peak (e.g., Steiner et al. 2013; Spencer et al. 2001). Makeig et al. (1999) identified four ICs in the ERP corresponding to a frontoparietal potential, a longer-latency parietal potential, a post-motor potential, and a left frontocentral potential.

### Aim

This study aims at unraveling aging effects on cognitive processes that are involved in signal detection tasks by identifying the independent sources that contribute to the P3 wave as observed in the recorded ERP. ICs derived from the complete sample of healthy participants will be reconstructed into single-subject EEG responses for each component. The sample is split in two age-groups, younger participants aged 18–46 years, and older participants, aged 46–82 years. The acquired single-trial EEGs will be compared between younger and older individuals in the sample. Based on our earlier work (Van Dinteren et al. 2014b), it is expected that one or more P3-ICs will emerge that differ between age groups, potentially reflecting compensatory brain activation (i.e. ICs with a more anterior topography). Further P3-ICs are expected that do not differ between age-groups (i.e. ICs with a more posterior topography) and rather reflect general task-related activation. Furthermore, exact low-resolution tomography analysis (eLORETA) (Pascual-Marqui 2007) is used to compare current source densities between younger and older participants for the P3-ICs to localize differences in cortical activity for every IC. The differences in cortical activity are expected to mainly originate from anterior regions, reflecting compensatory frontal activation in older participants.

## Methods

### Participants

The sample consisted of 99 healthy participants, aged 18 to 82 years, and all recruited and tested at the Research Institute Brainclinics (Nijmegen, The Netherlands). All participants voluntarily gave written informed consent. These data are a subset of the data reported in previous papers (Van Dinteren et al. 2014a, b, 2015). Exclusion criteria were a personal or family history of psychiatric disorders, neurological disorders, brain injuries, addiction or any other serious medical condition. Participants were required to refrain from caffeine, alcohol and nicotine for at least 2 hours prior to electroencephalographic data acquisition. The data collection and study protocol was approved by the local IRB (CMO Region Arnhem Nijmegen; #2002/008).

The sample was divided in a young and an old group with a cut-off at 46 years (Van Dinteren et al. 2014a). Sample demographics are shown in Table 1. The percentage of male/female participants did not significantly differ for the age-groups (*Χ* ^2^(1,99) = 0.529, *p* = .30). Additionally, post-hoc analysis of differences in global field power (GFP) by sex and by age-group revealed no significant differences at any frame in the degree of global cortical activation as reflected in scalp electrode recordings. GFP is a single measure that is developed by Lehmann and Skandries (1980) that is computed as the mean of all absolute potential differences in the scalp field corresponding to a spatial standard deviation, see Skrandies (1990) for more details.

**Table 1:** Demographics of the used sample

	N	Mean age (SD)	Males/females
Younger	50	23 (3.5)	19/30
Older	49	59 (9.6)	23/27
Total	99	41 (19.5)	42/57

### Experimental Paradigm

As part of a large international database study, participants completed a test battery containing both EEG and behavioral tasks. A description of the full test battery can be found in Williams et al. (2011). The paradigm with the main interest was the oddball paradigm. The oddball paradigm consisted of a quasi-random sequence of 280 frequent background tones (500 Hz) and 60 infrequent target tones (1000 Hz). No two targets could appear consecutively in trials. All stimuli (50 ms; 5 ms rise and fall time) were presented binaurally (via headphones) at a volume of 75dB SPL with an inter-stimulus interval of 1000 ms. Participants were instructed to press two buttons simultaneously (one for each index finger to counterbalance motor effects) when they heard a target tone and to ignore the background tones. Speed and accuracy of responses were both equally stressed in the instructions. Before the actual test, participants were presented with a brief practice run to clarify the distinction between the two tones.

We further analyzed reaction time (RT) data obtained within a continuous performance test (CPT) in which participants were required to press two buttons simultaneously when a letter on the screen was presented twice in a row in a series of 125 letters (B, C, D or G). The inter-stimulus interval was 2500 ms. Additionally, a go/no go paradigm was presented in which participants were presented with the word “push” in either red or green letters. They were required to push two buttons simultaneously when the word was presented in green letters and to withhold from pushing the buttons when the word was presented in red letters. A total of 168 stimuli was presented within the task with an inter-stimulus interval of 2000 ms.

### Electroencephalographic Data Acquisition

EEG acquisition was performed using a standardized methodology and platform (Brain Resource Ltd., Australia). Participants were seated in a sound and light-attenuated room, controlled at an ambient temperature of 22 °C. EEG data were acquired from 26 channels: Fp1, Fp2, F7, F3, Fz, F4, F8, FC3, FCz, FC4, T3, C3, Cz, C4, T4, CP3, CPz, CP4, T5, P3, Pz, P4, T6, O1, Oz and O2 (Compumedics Quick-Cap and NuAmps amplifier; 10–20 electrode international system) with a ground at AFz. Data were recorded reference-free and offline referenced to averaged mastoids. Horizontal eye movements were recorded with electrodes placed 1.5 cm lateral to the outer canthus of each eye (bipolar). Vertical eye movements were recorded with electrodes placed 3 mm above the middle of the left eyebrow and 1.5 cm below the middle of the left bottom eyelid. Skin resistance was <5 kOhms for all electrodes. A continuous acquisition was employed at a sampling rate of 500 Hz at all channels. A high cut-off filter at 100 Hz was employed prior to digitization.

### ERP Scoring

Conventional ERP averages were computed relative to the target and background stimuli for all EEG channels per participant using Brain Vision Analyzer (Brainproducts, Germany). Only segments with a correct target response were included in the target average. Before averaging, the EEG epochs were filtered with a high-pass IIR filter of 0.16 Hz (12 dB/Oct) and a low-pass filter of 40 Hz (24 dB/Oct). Vertical and horizontal ocular correction was applied according to the Gratton algorithm (Gratton et al. 1983). The segments ranged from −200 to 800 ms around stimulus presentation. Segments were DC detrended and baseline corrected for a pre-stimulus interval of −200 to 0 ms. Segments were considered to contain artifacts when there was a difference of 20 μV or more between two subsequent data points, when the difference between the highest and lowest voltage within a 100 ms epoch exceeded 150 μV, when the maximum or minimum amplitude respectively exceeded 100 or −100 μV within −200 to 300 ms, or when activity fluctuations were below 0.5 μV in a 50 ms time period. Rejected segments were not included in the calculation of the target average. There were 55 rejected target segments in the complete sample.

### Independent Component Extraction

To decompose the P3 into its constituent components, a group ICA on the multi-subject EEG data was set up (e.g., Eichele et al. 2011; Huster et al. 2015; Bridwell et al. 2014). Hence, after preprocessing of the EEG an adjusted number of background and target trials was randomly selected from each subject’s available data. Note that the number of trials has to be the same for each subject in order to compute a group ICA. The number of background trials for further processing was restricted such that the average noise level of the condition-specific single-subject ERPs, computed as root mean squares of the baseline periods, did not differ between the background and target conditions. This procedure was implemented not to bias the group ICA towards background-related brain responses. These constraints led to a selection of 250 trials (200 background and 50 target trials) from each of the 99 participants that entered the analysis.

Group ICA extracts statistically independent components consistently expressed across participants. In short, each single-subject dataset first undergoes an individual principal component analysis (PCA), thereby extracting most relevant and orthogonal time courses per subject. These first-level principal components then are used as variables in a second, group-level PCA, now estimating most-relevant and orthogonal principal component time courses capturing activity patterns correlated across subjects. These undergo ICA that finally computes the statistically independent component time courses. Importantly, this nested procedure is capable of capturing some of the topographical variability we usually see with EEG events found in each of the single-subject datasets of a multi-subject study. Please refer to Eichele et al. (2011) and Huster et al. (2015), for a more detailed description of the algorithm.

A total of ten independent components were extracted using group ICA, because two procedures suggested this to correspond to the intrinsic dimensionality of the data. First, the inspection of the single-subject PCAs revealed that on average ten components explained about 95% of the variance of the scalp EEG data. Second, the ICASSO software package was used to assess the reliability and stability of the ICA solutions of 100 runs (Himberg et al. 2004). This combination of group ICA and ICASSO was used for evaluating models with 9, 10, and 11 components, and the ten-component solution revealed sufficient reliability and stability. Correspondingly, at each level of this analysis, including single-subject PCAs, group PCA, and group ICA, ten components were extracted. The ten resulting group components are characterized by their topographies and time courses, with estimations aiming at a maximal statistical independence of the latter, and naturally lead to a characterization of the latent data structure at the group level.

Four of the ten ICs were selected for further and more detailed analyses based on their resemblance with the P3. This selection was made after inspection of the topographies and time courses of each component, and reflects the research question directed at the study of the differential frontalization of the P3 in young and old adults. To statistically analyze the differences between younger and older participants and to assess the brain sources underlying the selected independent components, the scalp EEG corresponding to each component was reconstructed for every single subject. Hence, for every subject the matrices estimated during the data reduction and group ICA steps were extracted and applied to the single-subject EEG. By means of these individual demixing matrices, the subject-specific component time courses and topographies were reconstructed that directly relate to the group-level components. Then, the subject-specific EEG for each component was reconstructed by multiplying each single component time course with its corresponding coefficients of the subject-specific mixing matrix. The subject-specific EEG patterns corresponding to certain independent components formed the basis for our statistical analyses. Group independent components as well as the subject-specific mixing and demixing matrices were computed using self-written MATLAB scripts that followed the procedures described in Eichele et al. (2011). In addition, EEGLAB’s implementation of Infomax ICA in runica() was used.

### Inverse Modeling

The subject-specific EEG/ERPs were reconstructed for every independent component and then compared between younger and older subjects. The data was entered in the eLORETA software (Pascual-Marqui 2002; Pascual-Marqui et al. 1994) that analyzes GFP of both groups at every time frame and produces a *t*-value for each time frame. Those time frames where the *t*-value exceeds the significance level (*p* < .01) comprise the ERP segments where the groups differ significantly from each other in GFP. These time frames were used in a spatial analysis. The software corrects for multiple testing by means of a randomization procedure.

The validated (Olbrich et al. 2009) eLORETA software yields images of current source density differences across the cortex. Current source density is calculated by squaring the weighed sum of scalp potentials for each voxel (A/m2). The computations are made in a realistic head model (Fuchs et al. 2002), using the MNI152 template (Mazziotta et al. 2001) with standard electrode positions displayed on the scalp (Jurcak et al. 2007; Oostenveld and Praamstra 2001). The three-dimensional solution space is restricted to cortical gray matter, as determined by the probabilistic Talairach atlas (Lancaster et al. 2000).

The eLORETA software solves the inverse problem under the assumption that the smoothest of all possible solutions (meaning that neighboring voxels should demonstrate maximally similar activation) is the most plausible one. This comes at the cost of low spatial resolution. Averaged current source density values for the two age groups were log-transformed and statistically compared. The software compares the groups on a voxel basis and identifies those voxels with *t*-values that exceed a significance level.

### Behavioral Analysis

Post-hoc, relationships between independent components and behavioral data from the oddball, CPT and go/no go paradigms were investigated by correlational analysis. To do so, peak amplitudes of the reconstructed independent component ERPs correlations with reaction times in the three paradigms were statistically assessed. A Bonferroni corrected alpha of 0.02 (initial alpha level of 0.05, three tests) was employed.

## Results

### ERP Time Frames

**Fig. 1:**
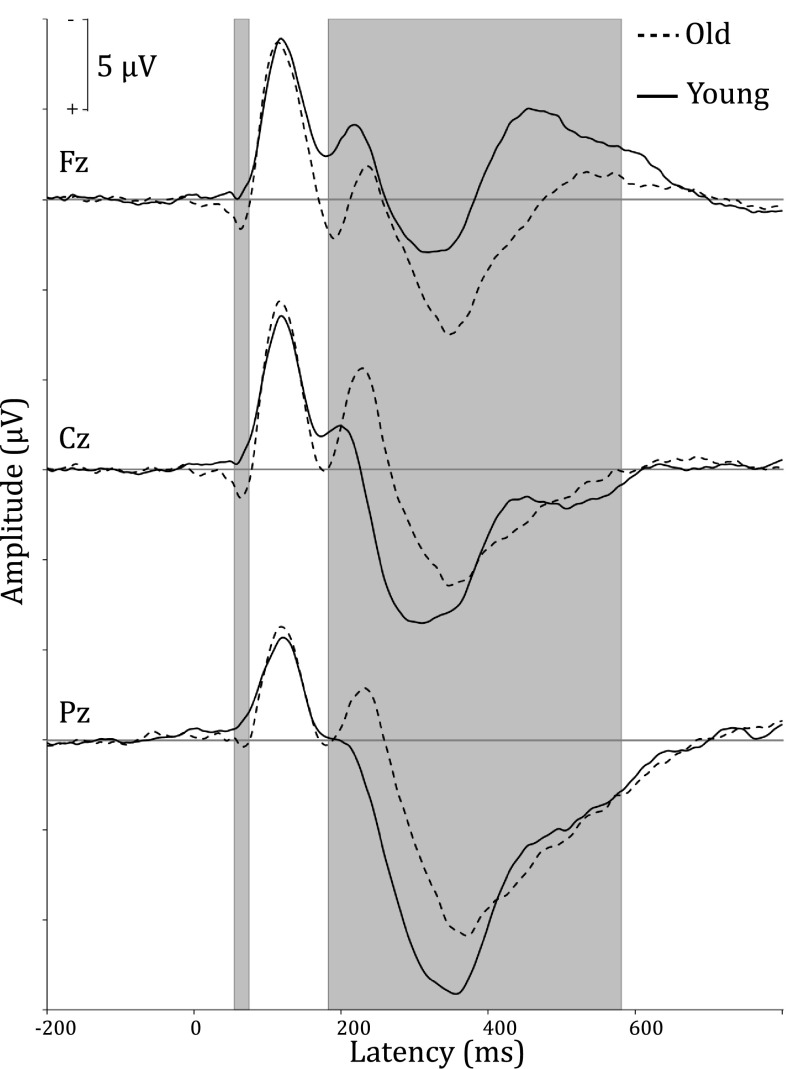
Grand average ERPs for younger and older participants at midline sites. *Grey* shading indicates significant group differences in global field power just after stimulus presentation and during the P3 interval (*p* < .01)

**Fig. 2:**
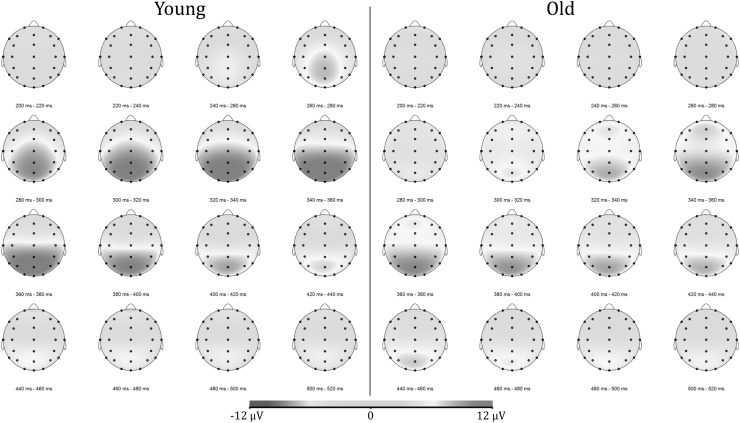
Head maps corresponding to the P3 time interval of 200–520 ms post-stimulus of younger and older participants. Notice the reduced activity at parietal electrodes, the higher activity at frontal electrodes, and the delayed onset in old participants compared to young participants

In Fig. 1 the grand average ERPs for younger and older participants at midline sites are shown. Figure 2 shows the head maps corresponding to the 200–520 ms post-stimulus time interval. The time frames when the difference in global field power between averaged group ERPs exceeded the significance level were at 54–74 and 182–580 ms (t > 4.953, *p* < .01).

### Independent Components

**Fig. 3:**
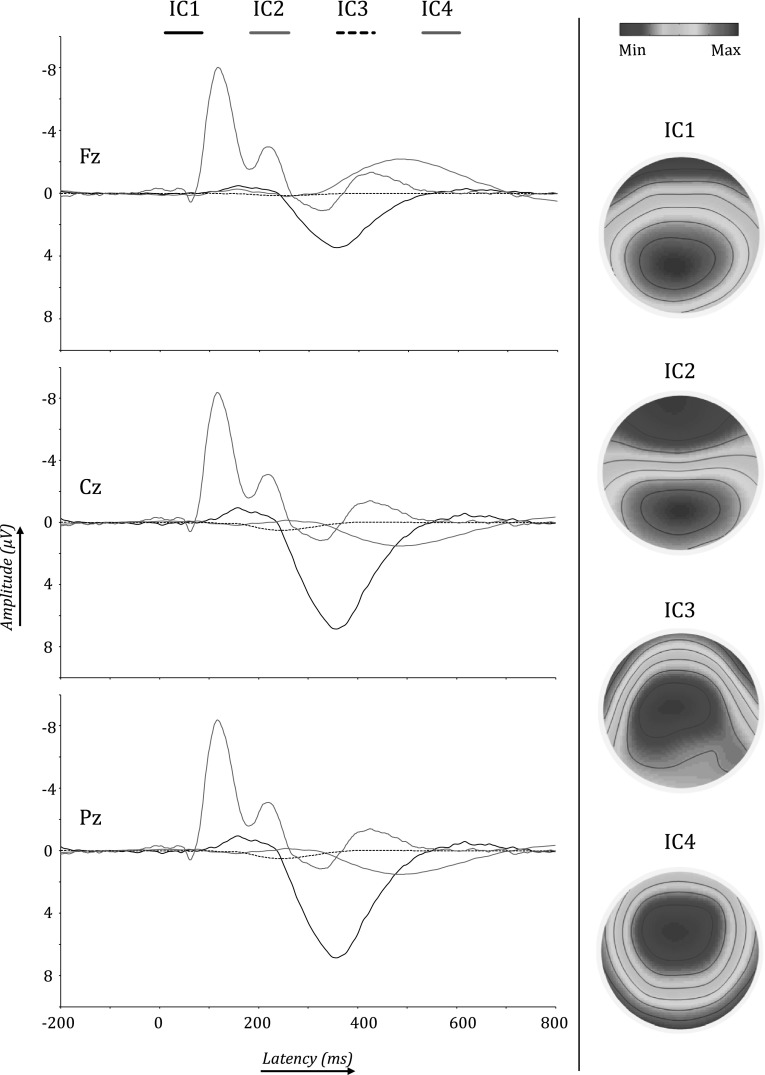
The *left half* of the figure shows ERPs of IC1, IC2, IC3 and IC4 at Fz, Cz and Pz. The *right half* of the figure shows the topographies of these four independent components. Topographies were scaled separately for each component to the global minimum and maximum values of the time series

**Fig. 4:**
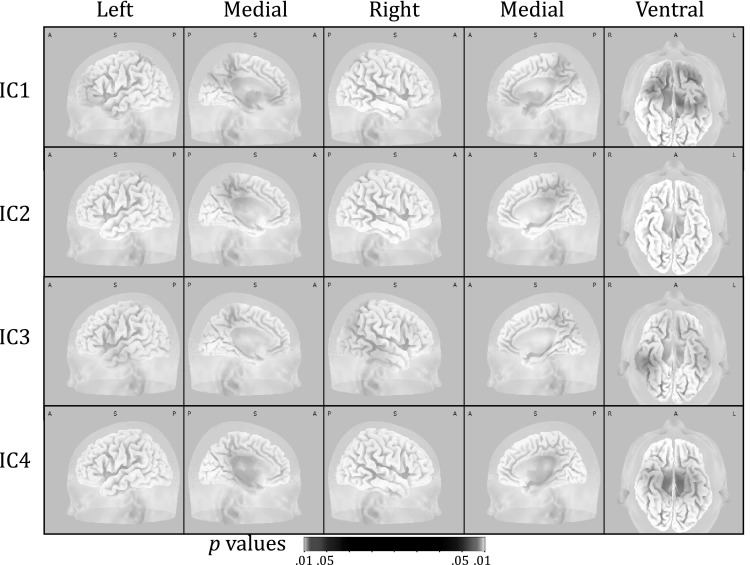
Different views of cortical sources of the four independent components (IC) that were derived from the P3 potentials of the complete sample

ICs are ordered according to their contribution to the variance of the EEG signal. Figures 3 and 4 depict the first four ICs for the complete sample. Using eLORETA the current source densities were calculated for localization purposes. IC1 was localized to an orbitofrontal/temporal as well as a medioparietal region; IC2 was localized to the precuneus/cingulate gyrus area; IC3 was localized to an area around the precuneus of the right parietal lobe; IC4 was localized to the posterior cingulate gyrus, see Fig. 4. Based on the ERP latency of IC4, this component seems to be related to earlier processing stages and is less likely related to the later higher order cognitive processes that the P3 represents.

### IC Group Comparisons

The ICs were subjected to a time-domain analysis using *t* tests to compare the two groups. For IC1, groups differed significantly from each other at 276–492 ms post-stimulus (t > 4.282, *p* < .01). For IC2, the groups differed significantly from each other at 208–250 ms post-stimulus (t > 4.266, *p* < .01). For IC3, the groups differed significantly from each other at 238–316 ms post-stimulus (t > 4.190, *p* < .01). Finally, for IC4, the groups differed significantly at 58–70 ms post-stimulus (t > 4.408, *p* < .01).

**Fig. 5:**
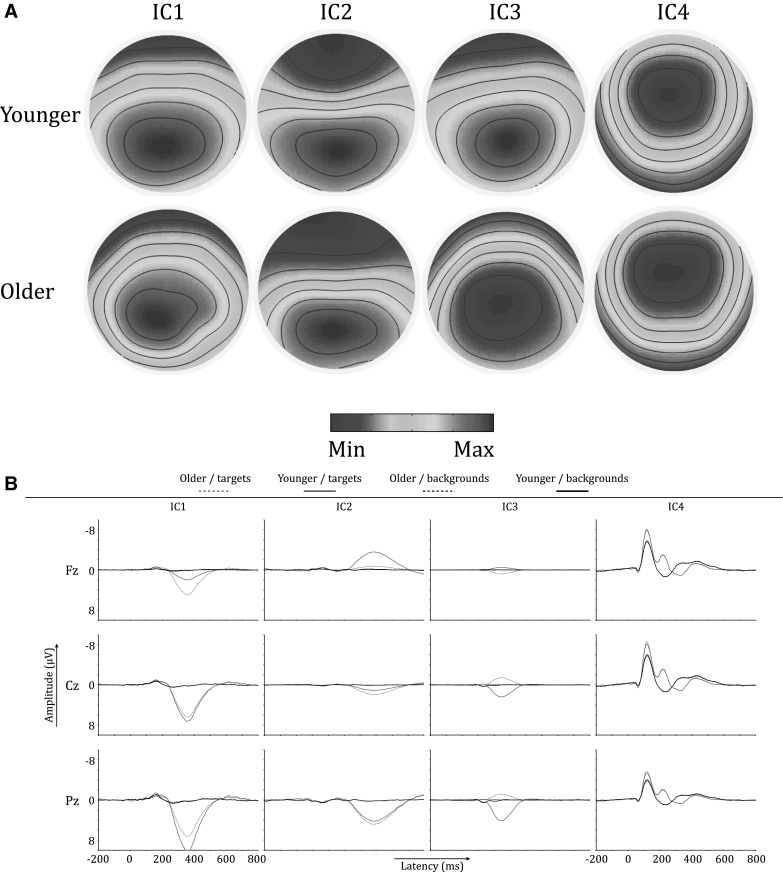
**a, b** Back-constructed ERPs of IC1, IC2, IC3 and IC4 for both age groups. **a** Shows topographies of the ICs for younger vs. older participants. **b** Shows ERPs of the independent components at Fz, Cz and Pz, for younger versus older participants. Topographies were scaled separately for each component to the global minimum and maximum values of the time series

**Fig. 6:**
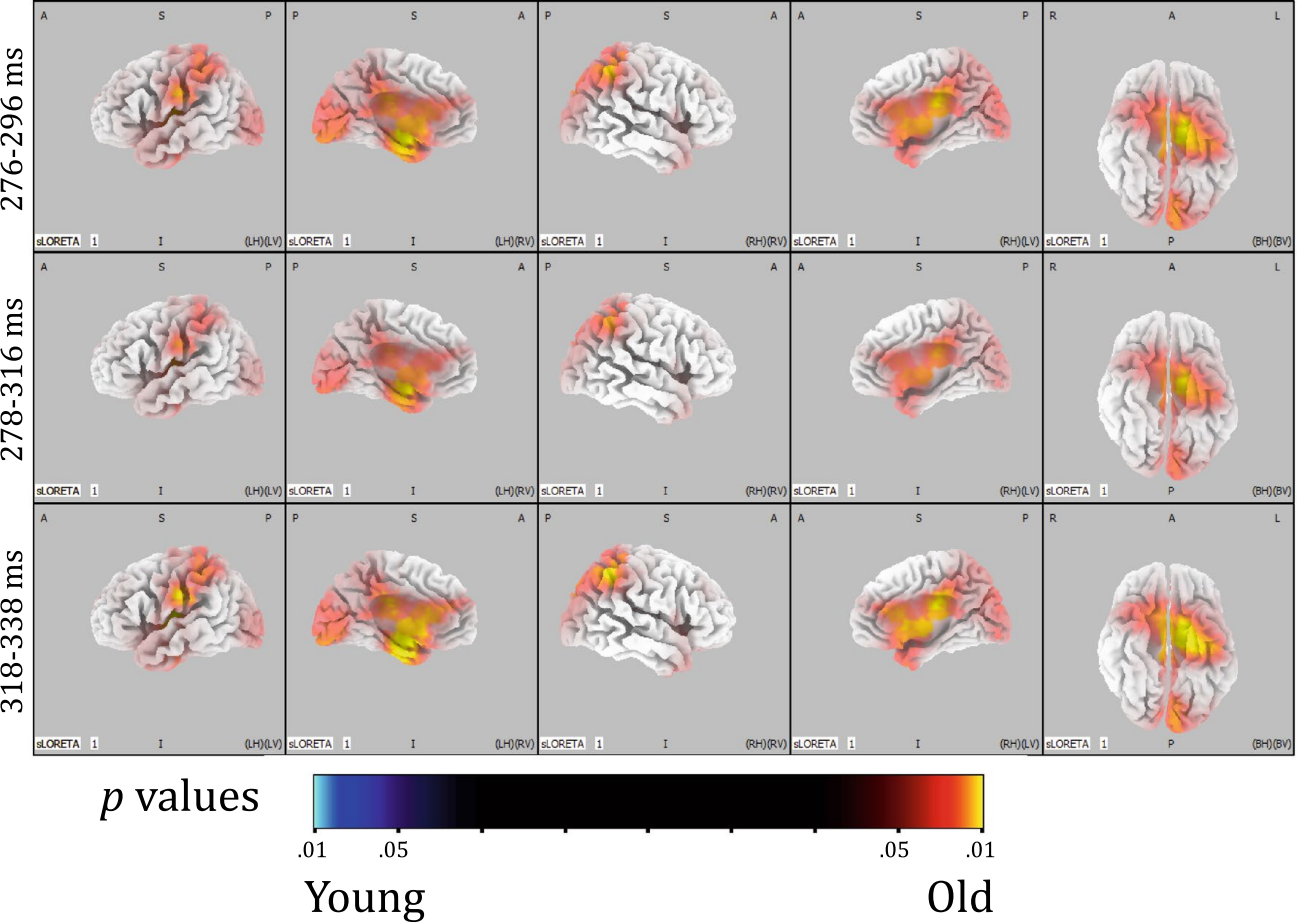
For IC1, older participants had significantly more activation than younger participants in the precentral gyrus and the parahippocampal gyrus. Difference in activation is depicted on a scale of *p* values. Voxels with more activation in older participants have a *red* color scheme. Voxels with more activation in younger participants have a *blue* color scheme (there are none). (Color figure online)

To test where in the brain these differences would be located, the specified time frames were segmented into 20 ms epochs in order to reduce the number of tests that were run per analysis. For every IC separately, segments were analyzed for group differences after source localization and by using voxel-wise *t* tests. Note that corrections for multiple comparisons are done by the eLORETA software. For IC1, older participants had significantly more activation in the precentral gyrus and the parahippocampal gyrus (PHG). This was true for the early part of IC1, i.e. 276–296 ms (t > 3.364, *p* < .01), 298–316 ms (t > 3.351, *p* < .01) and 318–338 ms (t > 3.499, *p* < .01). In the 318–338 ms epoch there were differences in cortical activations, besides the precentral gyrus and PHG, localized to the insula, cingulate gyrus, uncus, subcallosal gyrus, inferior parietal lobule and post-central gyrus. Figure 5a, b show the back-constructed ERPs of IC1 for both groups at Fz, Cz and Pz. It can be seen that IC1 displays a shift towards anterior electrodes in older participants. Figure 6 shows the differences between both groups in cortical activations for IC1. For IC2, IC3 and IC4, there were no brain regions indicating statistically significant differences between younger and older participants.

### Relation with Behavioral Data

Associations between individual peak amplitudes of the reconstructed IC1-ERP (at Pz) and RTs in the oddball paradigm, CPT, and the go/no go task were analyzed by non-parametrical correlational analysis.

**Fig. 7:**
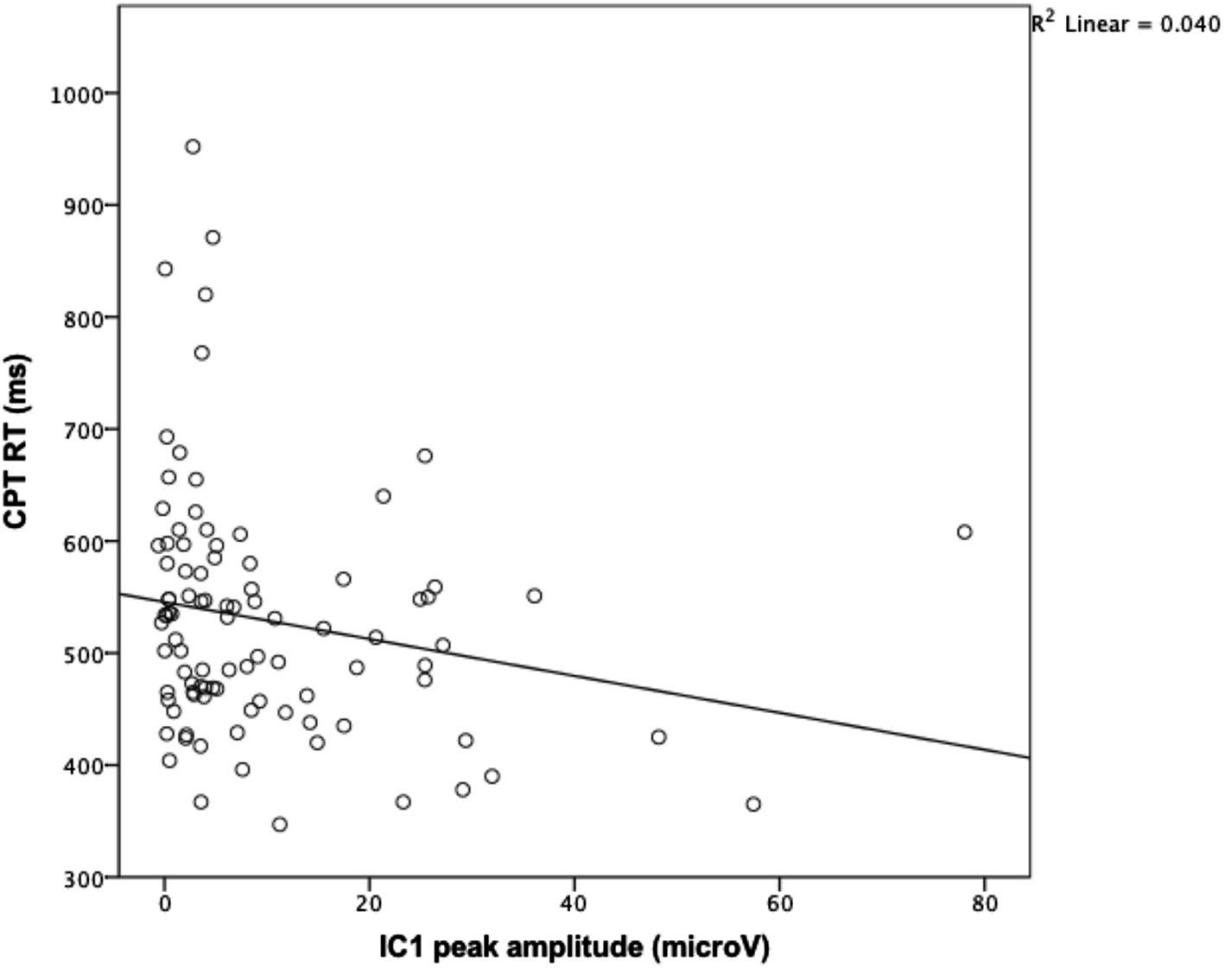
Higher peak amplitudes on reconstructed IC1 ERPs at Pz are associated with faster reaction times (RT) on a CPT task

There were no significant correlations between peak amplitudes and reaction times in the go/no go or oddball paradigms. However, peak amplitudes of the reconstructed IC1-ERPs correlated significantly with reaction times in the CPT (*r* = −252, *p* = .014, R^2^ = 0.06). Higher amplitudes were associated with faster reaction times, see Fig. 7.

Age group comparisons on the behavioral data using the Mann–Whitney U test demonstrate that median reaction times do not differ in the oddball and CPT tasks between younger and older participants. Median reaction times do significantly differ between younger and older participants in the go/no go task (Mann–Whitney U = 738, *N*_*young*_ = 49, *N*_*old*_ = 46, *p* = .004). The median reaction time of younger and older participants was 277 and 291 ms, respectively.

## Discussion

The aim of the present study was to unravel aging effects on oddball ERPs, and especially focusing on the P3 elicited in an auditory oddball paradigm. This was attempted by identifying the independent sources that contribute to the P3 and comparing these P3-ICs between younger and older participants. Furthermore, current source density reconstructions of the P3-ICs were compared between both groups to study differences in cortical activity. It was expected that some ICs reflect compensatory activation in older subjects, whereas other ICs reflect general task-related processes.

The ERPs of younger and older participants differed significantly in global field power in the P3 time frame, and to a lower degree also in an earlier time window. Based on their topography and temporal activity profiles, three ICs were identified as sources underlying the P3 wave (IC1, IC2 and IC3). Based on its ERP time course, IC4 appeared to be less likely P3-related, but because of its parieto-central topography it was also included in the initial analyses. For the whole group, IC1 was localized to an orbitofrontal/temporal and a medioparietal region; IC2 was localized to the precuneus/cingulate gyrus region; and IC3 was localized to a region around the right precuneus, see Fig. 4. Our results further corroborate earlier reports showing that the scalp P3 actually reflects a mixture of several statistically independent neural processes (Makeig et al. 1999).

Individual ERPs were back-reconstructed based on the ICs. The three IC-ERPs of the two age groups differed significantly in the time domain. In the source domain, only IC1 showed significant differences in cortical activity between older and younger participants. Here, older participants exhibited stronger activation than younger participants of precentral and parahippocampal regions. For the later part of the IC1 potential other cortical structures, i.e. the insula, cingulate gyrus, uncus, subcallosal gyrus, post-central gyrus and inferior parietal lobule, demonstrated significant differences in activation as well.

In sum, older participants use their available brain capacity differently from younger participants when they are performing the same oddball task. IC1 reflects a different mix of cortical sources in older participants compared to younger participants and therefore, this IC seems to be the most obvious P3-IC that partly might be reflecting compensatory processes in older age [i.e. the frontal P3 component we described earlier in Van Dinteren et al. (2014a)]. Differences in cortical activations were spread over multiple sources indicating there are possibly multiple neural networks involved. IC1 was localized to orbitofrontal and medial regions in the whole sample, which supports the idea that compensatory activation at least partially comes from frontal regions.

When the peaks from the reconstructed IC1 ERPs were compared with reaction times on the included tests a significant correlation was observed between IC1-ERP peak amplitudes and CPT reaction times. CPT performance has long been thought of as a test that assesses working memory but its construct validity has been questioned. The n-back task (CPT) typically demands more recognition where participants discriminate the target stimuli from similar stimuli, e.g. a specific letter among other letters in an array of stimuli (Kane et al. 2007). N-back task accuracy has been found to correlate with the Trail Making Test A, an information processing speed task (Miller et al. 2009). Therefore, IC1 may also be a component extracted from the ERP associated with speed of recognition processes. This altogether supports the assumption that this IC reflects the expected frontal compensation mechanisms, which are increasingly drawn upon during ageing.

There are some limitations to this study. Although, the oddball paradigm is very suitable for research with participants at different ages because of its simplicity, a more difficult paradigm may be even better suited to test for compensatory effects in older ages. A paradigm that allows for manipulation of the task load would be a valuable addition in future research. Another limitation is that a 26-channel EEG is relatively limited for inverse modelling purposes. Although eLORETA has been validated in multiple studies and the standard 10/20 EEG system employing electrode montages with for example 25 electrodes has been proven sufficient for source localization (Pascual-Marqui et al. 2002), ideally more channels should be used to increase the accuracy of localizations (Lantz et al. 2003; Srinivasan et al.; Michel et al. 2004).

In conclusion, the P3 can be separated into three independent components. All of these components differ regarding the strength of activity between younger and older participants when assessed in the electrode space. Despite the significant differences found in the time-domain the effects were not present in the source localization for all ICs, which may of course result from the necessity to correct test statistics for multiple comparisons However, the cortical sources of one component differed between younger and older participants and source analyses of the back-reconstructed ERPs indicated more frontal activation and lesser parietal activation in older participants compared to younger participants. This IC possibly reflects compensatory brain activation that is increasingly recruited as people age. In favor of CRUNCH, as people age the sources of IC1 are shifting whereby frontal contributions are increasing and parietal contributions are declining.
